# Development of a noninvasive device and method for measuring immune parameters in skin disease

**DOI:** 10.1016/j.xjidi.2026.100456

**Published:** 2026-02-03

**Authors:** Eveline D. de Geus, Sem Koornneef, Nalini C. Sreeram, Robbert J. Rottier, H. Bing Thio, Willem A. Dik

**Affiliations:** 1 Department of Dermatology, Erasmus MC, University Medical Center, Rotterdam, The Netherlands; 2 Department of Pediatric Surgery, Erasmus MC-Sophia, Rotterdam, The Netherlands; 3 Department of Cell and Developmental Biology, Erasmus University Medical Center, Rotterdam, The Netherlands; 4 Laboratory Medical Immunology, Department of Immunology, Erasmus MC, University Medical Center, Rotterdam, The Netherlands; 5 Laboratory Medical Immunology, Reinier Haga Medisch Diagnostisch Centrum (RHMDC), Delft, The Netherlands

**Keywords:** Atopic dermatitis, Diagnostics, Monitoring, Noninvasion device, Psoriasis

## Abstract

Diagnosis, treatment, and monitoring of skin disease, including psoriasis and atopic dermatitis (AD), currently depend on clinical evaluation. Access to easily accessible and reliable biomarkers would facilitate diagnosis, treatment, and monitoring of disease activity. Currently used methods to collect biomarkers from the skin include taking biopsies, tape stripping, and collecting suction blister fluid. These methods cause discomfort and are not suitable for longitudinal monitoring. In this paper, we designed a skin wash device and method to noninvasively collect biomarkers from skin. We validated our device and method by application of recombinant cytokines on skin of healthy individuals. Thereafter, we determined the skin inflammatory profiles in patients with AD and patients with psoriasis and compared these with those of healthy control volunteers. We show that with our method, elevated concentrations of inflammatory cytokines were detected in samples obtained from patients with AD or psoriasis, and we were able to distinguish between these 2 patient groups on the basis of their cytokine profiles. We propose that our skin wash device and method is highly promising to improve the diagnostics, treatment, and monitoring of skin diseases. Furthermore, it could be used in other research areas leading to more insight in the (immune)pathogenesis of skin diseases.

## Introduction

Diagnosis and treatment of prevalent skin conditions, including psoriasis and atopic dermatitis (AD), depend on clinical evaluation, and this hampers treatment. Access to easily obtainable, reliable biomarkers would not only facilitate diagnosis but could also be used to determine optimal treatment, to monitor the response to treatment, and to assess disease activity. Potential biomarkers for different skin diseases have been analyzed in serum; however, especially in mild conditions, disease-related biomarkers may not be upregulated in serum (He et al, 2021b). Currently used methods to measure biomarkers in the skin include taking biopsies, tape stripping, and collecting suction blister fluid. These methods cause discomfort and are not suitable for repeated analysis of a patient’s skin. Tape stripping has been used for the determination of a broad spectrum of biomarkers. The technique causes only mild discomfort when performed on healthy skin but can be more painful on lesional skin. Tape stripping mechanically disrupts the epidermal barrier, leading to transient inflammation, resulting in microvascular changes in the skin (He et al, 2021a). The production of a suction blister causes moderate discomfort in healthy individuals, with slight pain and itching. Furthermore, suction blister fluid can be contaminated with blood (Sjöbom et al, 2020).

We developed a device and method for noninvasive measurement of inflammatory biomarkers directly from skin. The method is painless and does not induce skin damage, making it well-suited for longitudinal monitoring. As a proof of principle, we focused on 2 pathological skin conditions that differ in underlying immune pathogenesis and associated cytokine/chemokine profiles: (i) AD, a T helper (Th)2–driven condition (Boothe et al, 2017; He et al, 2021a; Schuler et al, 2023; Zhang et al, 2023), and (ii) psoriasis, a Th17-driven condition (He et al., 2021a; Ramessur et al, 2022; Zhang et al, 2023).

In this paper, we describe our skin wash device and method to reliably measure cytokines, chemokines, and other immune mediators in skin wash samples.

## Results

### Detection of recombinant cytokines applied to healthy skin

**Figure 1:**
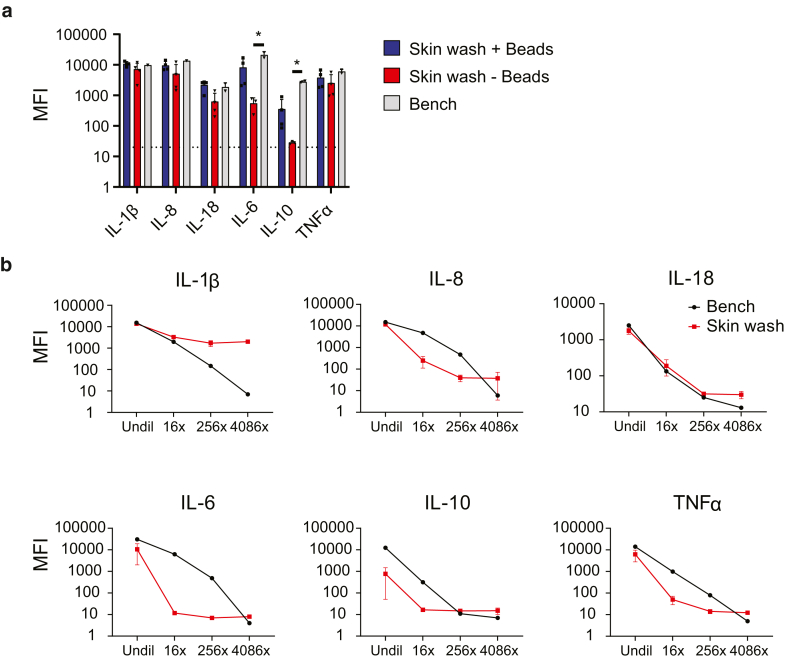
**Retrieval of spiked recombinant protein.** Retrieval of spiked protein applied at 10 μg/ml is shown. (**a**) The dotted line depicts a blank measurement. Shown is a retrieval of spiked protein applied at a ×16 serial dilution, starting at 10 μg/ml. (**b**) Red line: recombinant protein mix applied to the forearm; black line: recombinant protein mix left on the bench for the duration of the skin sampling procedure. Data are depicted as mean ± SD of 4 individual measurements where possible. ∗*P* < .05.

We first assessed recovery of recombinant cytokines applied to healthy control (HC) skin. Signals for IL-1β, IL-8, IL-18, and TNFα did not differ between the control mix left on the bench, wash buffer with beads, and wash buffer without capture beads, indicating that these cytokines were efficiently eluted from the skin. We found that recovery of IL-6 and IL-10 applied to the healthy skin was significantly improved when cytokine-specific Luminex capture beads were added to the wash buffer in the sampling device (Figure 1a); we consider this likely to be related to preventing protein degradation by local proteases. The capture beads are 6.5 μm in size and will therefore not pass through the nylon membrane, preventing direct contact with the skin. Capture beads were routinely added to wash buffer from here onward.

Serial dilution of recombinant proteins applied to the skin was associated with decreased detection of most cytokines, as expected (Figure 1b). The exception was IL-1β, which leveled off from 16× dilution onward. This could be related to detection of skin-endogenous IL-1β derived from keratinocytes and skin-resident immune cells (eg, owing to *Staphylococcus aureus* colonization [Lang et al, 2024]). These data demonstrate that our method can reliably detect even low cytokine concentrations.

### Detection of endogenous cytokines, chemokines, and soluble receptors from skin of healthy volunteers and patients with AD or psoriasis

**Figure 2:**
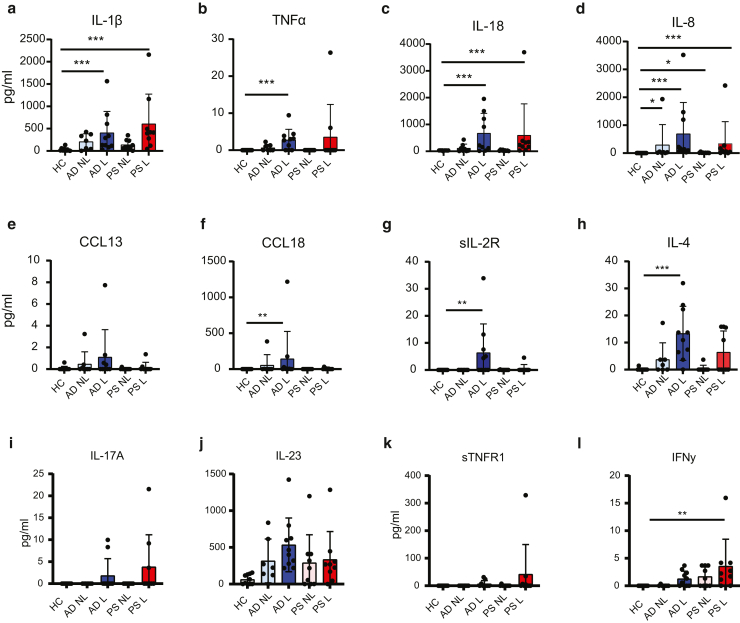
**Detection of inflammatory cytokines in skin wash of healthy volunteers and patients with AD and psoriasis.** Cytokine concentrations were measured in skin wash fluid using Luminex assay. Inflammatory cytokines (**a**) IL-1β, (**b**) TNFα, (**c**) IL-18, and (**d**) IL-8. AD-related immune mediators (**e**) CCL13, (**f**) CCL18, (**g**) sIL-2R, and (**h**) IL-4. Psoriasis-associated immune mediators (**i**) IL-17A, (**j**) IL-23, (**k**) sTNFR1, and (**l**) IFNγ. Concentrations are depicted as mean ± SD, with individual data points shown. PS denotes psoriasis, NL denotes nonlesional, and L denotes lesional. ∗*P* < .05, ∗∗*P* < .01, and ∗∗∗*P* < .001. AD, atopic dermatitis; HC, healthy control; sIL-2R, soluble IL-2R; sTNFR1, soluble TNFR1.

We then analyzed endogenous inflammatory cytokines in skin wash from HC and lesional and nonlesional skin of patients with AD and psoriasis (demographics are presented in Table 1). None of the volunteers reported any adverse effects after wearing the device. IL-1β was detected in 9 of 10 HC samples (Figure 2a). Concentrations of IL-1β were significantly higher in lesional samples of patients with AD and psoriasis but not in the nonlesional skin (Figure 2a). TNFα, IL-18, and IL-8 were not detected in HC. TNFα was significantly elevated in AD lesional skin, whereas IL-18 was elevated in lesional skin of both patients with AD and psoriasis (Figure 2b–d). IL-8 concentrations in AD and psoriasis nonlesional samples were elevated and were even higher in lesional skin (Figure 2d).

**Table 1:** Demographics of Healthy Controls and Patients

Demographics	Healthy Control (n = 10)	AD(n = 10)	Psoriasis (n = 10)
Age, y, average (range)	43 (24–60)	34.9 (24–61)	53.1 (31–84)
Female, %	70%	70%	40%
Itch score (0–10), average (range)	0	5.5 (1–10)	3.2 (1–9)

Next, we examined the expression of AD-associated inflammatory molecules. CCL13 tended to be elevated in AD lesional skin, but this was not significant (Figure 2e). CCL18 and soluble IL-2R (sIL-2R) were significantly increased in AD lesional skin, although these mediators were only detected in a subset of patients (6 of 10 for CCL18 and 5 of 10 for sIL-2R) (Figure 2f and g). IL-4 tended to be increased in nonlesional AD skin and was significantly increased in lesional AD skin (Figure 2h). These proteins were low to nondetectable in HC and psoriasis samples, with the exception of IL-4, which was detected at low concentration in a subset of patients with psoriasis (3 of 10).

**Figure 3:**
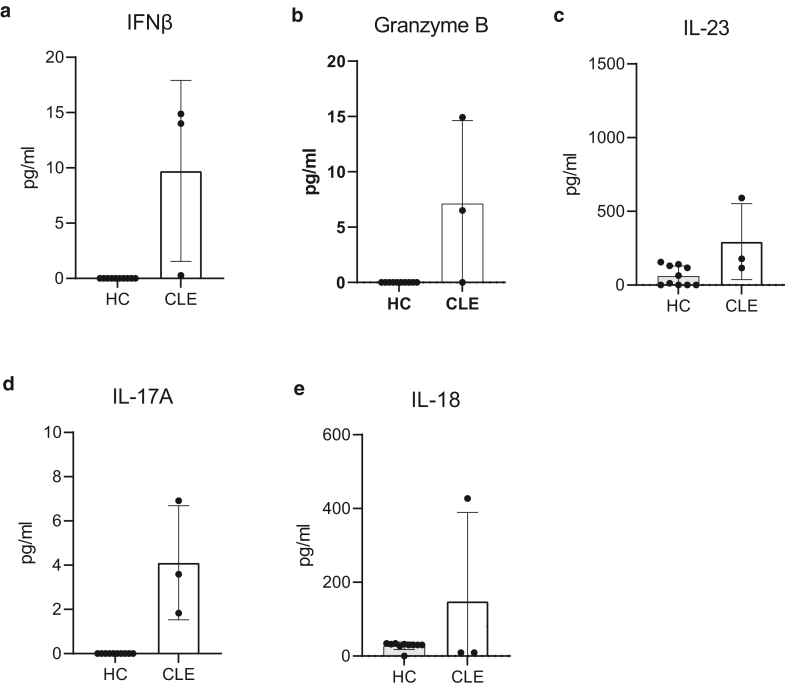
**Detection of CLE-associated immune mediators in skin wash of healthy volunteers and patients with CLE.** Cytokine concentrations were measured in skin wash fluid using Luminex assay. We collected 1 lesional and 1 nonlesional sample from patient 1 and a nonlesional skin wash sample from patient 2. (**a**) IFNβ. (**b**) Granzyme B. (**c**) IL-23. (**d**) IL-17A. (**e**) IL-18. Concentrations are graphed as mean ± SD. CLE, cutaneous lupus erythematosus.

We also the explored inflammatory molecules related to psoriasis (Figure 2i–l). IL-17A and soluble TNFR1 were only detected in lesional skin in 2 of 10 patients with psoriasis but also in 2 of 10 patients with AD, whereas it was absent in HC. IL-23 was readily detected in both lesional and nonlesional skin of both patients with AD and psoriasis, without differences between groups. Although no statistical significance was reached, IL-23 concentrations were higher in both AD and psoriasis samples than in control samples. IFNγ concentrations were significantly elevated in lesional skin samples from psoriasis (Figure 3l). Low concentrations of IFNγ were also detected in psoriasis nonlesional and AD lesional skin.

### Skin wash concentrations of IL-8, IL-4, and IFNγ correlate with clinical severity

Biomarkers predictive for clinical severity and/or predictive for flare ups of disease would enhance treatment selection and monitoring. To determine whether detected cytokines correlated with disease severity, we performed Spearman correlations. Both lesional and nonlesional IL-8 concentrations showed strong correlations with patient-reported itch scores in both patients with AD and psoriasis (Table 2). Both IL-4 concentrations in AD and IFNγ concentrations in psoriasis lesional skin wash but not in nonlesional skin showed strong correlations with subjective itch score (Table 2). The observed correlations between these cytokines with itch scores underscore the clinical relevance of our method. Skin IL-8 may represent a marker of subclinical inflammation, given its elevation in nonlesional skin.

**Table 2:** Correlation of Cytokine Concentrations and Subjective Itch Score

	Itch	L IL-8	NL IL-8	L IL-4	NL IL-4	L IFNγ	NL IFNγ		
Itch	1								
L IL-8	0.895∗∗	1							**Legend:**
NL IL-8	0.858∗∗	0.856∗∗	1						1
L IL-4	0.719∗∗	0.634∗∗	0.724∗∗	1					0,5
NL IL-4	0.332	0.496∗	0.384	0.429∗	1				0
L IFNγ	0.731∗∗	0.698∗∗	0.763∗∗	0.66∗	0.452	1			−0,5
NL IFNγ	0.567	0.351	0.351	0.018	-0.148	0.592∗	1		−1

Spearman's rank correlation coefficients were determined using concentrations of IL-8, IL-4, and IFNγ in lesional (denoted as L) and nonlesional (denoted as NL) skin of patients with AD and psoriasis and using itch scores. ∗*P* < .05 and ∗∗*P* < .01.

## Discussion and Potential Applications

Currently, no accessible and reliable biochemical biomarkers exist for diagnosis and monitoring of disease severity and treatment response for prevalent skin diseases such as but not limited to AD and psoriasis. For AD, most diagnostic criteria are currently based on cutaneous signs of atopy (Hanifin and Rajka, 1980), and disease severity can be assessed using the Eczema Area and Severity Index or SCOring Atopic Dermatitis scoring systems (Barbier et al, 2004, 2004; Kunz et al, 1997). Similarly, the diagnosis of psoriasis is clinical, on the basis of patient history and morphology and distribution of skin lesions (reviewed in Griffiths et al, 2021). Psoriasis severity is often assessed using the PASI (Puzenat et al, 2010). For both AD and psoriasis, clinical assessment depends on the clinician’s experience, and this can result in variation in clinical severity assessment. Having access to noninvasively collectable biomarkers would greatly facilitate diagnosis, treatment, and monitoring of prevalent skin diseases such as but not limited to AD and psoriasis (Libon et al, 2024; Mortlock et al, 2023).

In this paper, we present a skin wash sampling device and detection method that allows for reliable detection of immune mediators associated with inflammatory skin diseases in a noninvasive manner. We solved issues with protein degradation by adding a membrane to the device, allowing the use of capture beads. This way, proteins are more reliably detected, whereas the capture beads cannot come in direct contact with the skin. This is especially important in skin with reduced barrier integrity, as reported in inflammatory skin diseases, including AD and psoriasis (Orsmond et al, 2021; van den Bogaard et al, 2023). The method is highly flexible because capture beads can be chosen for different proteins of interest. For this study, we used a panel of cytokines associated with either AD or psoriasis, and our method reliably distinguished these 2 skin diseases. In addition, our method is suited for repeated sampling of the skin because it does not cause discomfort nor damages the skin.

Biomarkers predictive for clinical severity and/or predictive for flare ups of disease would enhance treatment selection and monitoring. We not only observed an increase in Th2 cytokines and chemokines for AD and in IFNγ for psoriasis but also observed elevated IL-8 concentrations in nonlesional skin of these patients when compared with the skin of HC volunteers. Moreover, concentrations of the above-mentioned cytokines correlated well with subjective itch scores. Further research should focus on investigating (combinations of) markers, including IL-8, predictive for exacerbation of skin disease.

Besides facilitating diagnosis and monitoring of inflammatory skin diseases, such as AD and psoriasis as studied in this paper, we propose that this method has wider implications. Indeed, we performed a pilot study collecting skin wash samples from 2 patients with cutaneous lupus erythematosus (CLE), a condition driven by type I IFN pathway activation (Rodríguez-Carrio et al, 2023). In these samples, without sufficient power for statistical testing, we detected elevated concentrations of IFNβ, granzyme B, IL-23, IL-17A, and IL-8 compared with those in HCs (Figure 3). Although we only included 2 patients to date, these findings underline that our device has applications beyond skin disease only.

Our method yielded similar immune profiles as already published results. However, our samples are procured in a noninvasive manner and do not require downstream isolation and elution steps prior to analysis. Results from tape stripping experiments were highly comparable with our results, with increased concentrations of IL-18, IL-1β, IL-8, TNFα, and *IL4* in tape stripping material of AD lesional skin (He et al, 2021a, 2020) and increased *IL1B* and *IFNG* in material collected from psoriasis lesional skin (He et al, 2021a). Most literature describing immune parameters in sweat focused on HC volunteers. In both passive and exercise-induced sweat, IL-1α and IL-1β were detected (Dai et al, 2013; King et al, 2023; Marques-Deak et al, 2006), similar to our observations. Low concentrations of IL-6 and TNFα were also described (Dai et al, 2013; Hladek et al, 2018). We did not detect these cytokines in HC volunteers, likely owing to differences in analysis methods (recycling immunoaffinity chromatography vs multiplex Luminex assay). Portugal-Cohen et al (2024, 2013, 2012, 2011) used a relatively similar skin wash sampler, yet without addition of multiplex Luminex beads. They analyzed IL-1α, TNFα, and IL-6 in healthy, AD, and psoriasis skin. Contrary to our data, they did not observe significant differences in TNFα concentrations between control skin and AD skin; this discrepancy may well be caused by cytokine degradation during the sampling procedure, especially because inflamed skin has increased protease activity (Nomura et al, 2020; Xiao et al, 2024).

In summary, we designed and validated a skin wash device and sampling method and showed that these can be used to reliably detect cytokine profiles associated with AD or psoriasis. On the basis of our preliminary results in patients with CLE, we have indications that our device has applications beyond inflammatory skin disease. We propose that our skin wash device and sampling method is a highly promising approach to skin disease diagnostics and monitoring and can be used in research into immunopathological mechanisms of skin disease and systemic inflammatory diseases.

## Materials and Methods

### Ethical approval

This study was conducted according to the principles of the Declaration of Helsinki and in accordance with the national Medical Research Involving Human Subjects Act. The study was approved by the Medical Ethics Committee of Erasmus MC Rotterdam (MEC-2024-0322). The study was registered in the overview of medical research in The Netherlands (NL-OMON57070).

### Study design, recruitment, and study population

This prospective cohort study was conducted at the dermatology clinic and the Laboratory Medical Immunology at Erasmus MC (Rotterdam, The Netherlands). Healthy volunteers were recruited from employees of the Department of Immunology through a general email. Patients with AD or psoriasis from the dermatology clinic were approached by phone call or during routine visits. All potential volunteers were sent the information forms and were given ample time to decide to participate. Before study procedures started, all participants gave written informed consent. Inclusion and exclusion criteria are shown in Table 3 (HCs) and Table 4 (patients). Volunteers did not apply cosmetics or any topical treatment to the sampling site on the day of sampling.

**Table 3:** Inclusion and Exclusion Criteria for Healthy Controls

Inclusion Criteria for Healthy Controls	Exclusion Criteria for Health Controls
Immunologically healthy	Immunological disease, (seasonal) allergy
Nonobese	Obese
No malignant disease	Malignant disease
Not prescription medication (with the exception of oral contraceptives)	Under direct supervision of the principal or coordinating investigator or a student/intern
Aged >18 y

**Table 4:** Inclusion and Exclusion Criteria for Patients

Inclusion Criteria for Patients	Exclusion Criteria for Patients
Diagnosed with AD, psoriasis	Malignant disease
Nonobese	Obese
Aged >18 y	

Abbreviation: AD, atopic dermatitis.

### Skin wash device printing and assembly

The skin wash device was designed with Fusion 360 (AutoDesk, student licence) and converted to .stl format for printing. The .stl file of the well and lid for the skin wash device were uploaded to the PreForm (Formlabs) software and printed at a resolution of 0.025 mm (2.75 h; resin used: 3 ml). The well of the skin wash device was tilted and included supports with a density of 0.7 and touching points of 0.35 mm. The lid was directly printed on the Build Platform facing upward. The skin wash device was printed using a Form 3B+ printer (FormLabs), Clear resin V4 (FormLabs), and a Tank V2.1 (FormLabs).

**Figure 4:**
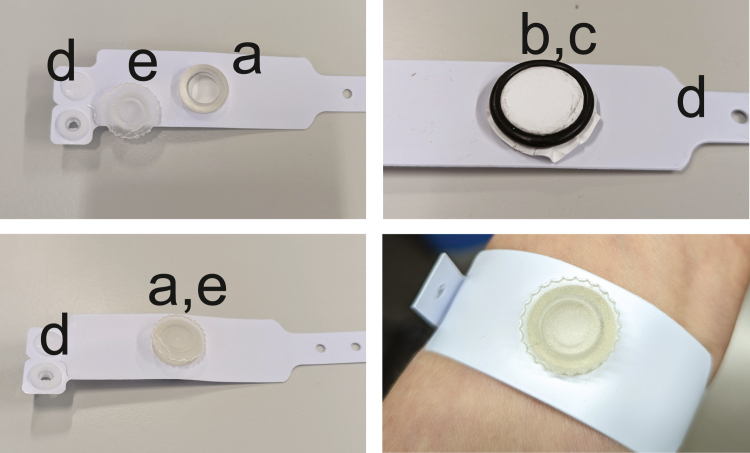
**The skin wash device.** (**a**) Custom-made 3D printed well, with (**b**) a 0.8-μm pore size nylon membrane attached with (**c**) an O ring. (**d**) The well is attached to the arm using an adjustable bracelet. (**e**) The well is closed with a screw cap. The bottom right panel shows one of the authors wearing the device. 3D, 3-dimensional.

Once printed, the device was washed in 100% isopropanol alcohol using the Form Wash (FormLabs) for 20 minutes. Next, the supports were removed with a scalpel and further air dried with compressed air. Finally, the device was cured with UV light using the Form Cure (FormLabs) at 60 °C for 60 minutes. Before use, a nylon membrane with a 0.8-μm pore size (Cytiva) was attached to the bottom of the well using a rubber O-ring and cut to size. A hospital bracelet with a hole matching the diameter of the well was used to keep the well in place (Figure 4).

### Detection of recombinant human cytokines applied to skin

*E coli*–derived recombinant human TNFα, IL-1β, IL-6, IL-8, IL-10, and IL-18 (endotoxin levels <0.10 EU/μg by the limulus amebocyte lysate method) were purchased from R&D Systems and reconstituted in PBS. A mix was made with 10 μg/ml of each recombinant cytokine, and 10 μl was pipetted onto the skin of the forearm of a healthy volunteer. After air drying, the well of the skin wash device was placed on the skin, 300 μl cold PBS/0.05% Tween-20 ± 10 μl containing Luminex capture beads specific for the applied proteins (R&D Systems, Bio-Techne) was added to each well, and the screw cap closed. After 30 minutes, the screw cap was removed, and the fluid was collected from the well. The bracelet was then removed, and the device was disassembled. The membrane was added to the vial containing the collected fluid, with the edge squeezed between the lid of the vial. Samples were directly processed for analysis and not stored beforehand. Samples were centrifuged for 5 minutes at 10,000*g*, and the collected fluid was processed directly for Luminex assay as per the manufacturer’s instructions. Cytokine signal intensities detected from skin wash fluid were compared with the signal intensities in the original cytokine mixture that was left on the bench at room temperature for the time period of the whole skin wash procedure.

### Detection of endogenous cytokines, chemokines, and soluble receptors from skin

One sample was taken from HC volunteers by placing a well on the forearm. Two samples were taken from patients—1 from nonlesional skin and the second from lesional skin—by placing a well on each of the areas. Placement was restricted to the forearm. Sampling and processing were performed as described earlier. To prevent contamination of the sample, the researchers wore gloves during the sampling procedure. As a proxy for clinical severity, patients were asked to score their level of itch out of 10.

### Luminex assay

A total of 14 inflammatory markers, reflecting general inflammation, cytokines, and chemokines associated with AD and/or psoriasis, were selected (Table 5). These biomarkers were measured in skin wash samples using a Customized Luminex human cytokine multiplex panel, according to the manufacturer’s instructions (R&D Systems, Bio-Techne).

**Table 5:** Inflammatory Molecules Selected for Multiplex Analysis

AD	Psoriasis	General Inflammation
CCL13	IL-17A	TNFα
CCL18	IL-23	IL-6
sIL-2R	IFNγ	IL-8
IL-4	sTNFR1	IL-1β
		IL-18 IL-10

Abbreviation: AD, atopic dermatitis; sIL-2R, soluble IL-2R; sTNFR1, soluble TNFR1.

### Data analysis

Graphs were prepared with GraphPad Prism 9.0.0. Statistical analyses were performed with the Statistical Package for the Social Sciences (version 28), using the Kruskal–Wallis tests with posthoc correction for multiple comparisons. Correlations were determined using Spearman's rank correlation. A *P* ≤ .05 was considered statistically significant. Youden's indices were only reported above 0.50 because a value below 0.50 does not meet the empirical standards to contribute as a potential diagnostic test.

## Ethics Statement

This study was conducted according to the principles of the Declaration of Helsinki and in accordance with the national Medical Research Involving Human Subjects Act. The study was approved by the Medical Ethics Committee of Erasmus MC Rotterdam (MEC-2024-0322). The study was registered in the overview of medical research in The Netherlands (NL-OMON57070). All potential volunteers were sent the information forms and were given ample time to decide to participate. Before study procedures started, all participants gave written informed consent.

## Data Availability Statement

The data that support the findings of this study are available from the corresponding author upon reasonable request.

## ORCIDs

Eveline D. de Geus: http://orcid.org/0000-0003-4453-3805

Sem Koornneef: http://orcid.org/0009-0002-8166-9234

Robbert J. Rottier: http://orcid.org/0000-0002-9291-4971

H. Bing Thio: http://orcid.org/0000-0001-5095-4281

Willem A. Dik: http://orcid.org/0000-0001-5235-3156

## Conflict of Interest

A patent application covering the technology described in this publication has been submitted by authors EDdG and WAD, also inventors on such application. The patent application is currently pending. HBT is an European Academy of Dermatology and Venereology board member. The remaining authors state no conflict of interest.
